# Association between bronchopulmonary dysplasia and death or neurodevelopmental impairment at 3 years in preterm infants without severe brain injury

**DOI:** 10.3389/fneur.2023.1292372

**Published:** 2023-11-15

**Authors:** Wenli Li, Yong Wang, Juan Song, Chen Zhang, Yiran Xu, Falin Xu, Xiaoyang Wang, Changlian Zhu

**Affiliations:** ^1^Henan Key Laboratory of Child Brain Injury and Henan Pediatric Clinical Research Center, Third Affiliated Hospital and Institute of Neuroscience, Zhengzhou University, Zhengzhou, China; ^2^Department of Neonatology, Third Affiliated Hospital of Zhengzhou University, Zhengzhou, China; ^3^Center for Perinatal Medicine and Health, Institute of Neuroscience and Physiology, Sahlgrenska Academy, University of Gothenburg, Gothenburg, Sweden; ^4^Department of Women’s and Children’s Health, Karolinska Institute, Stockholm, Sweden; ^5^Center for Brain Repair and Rehabilitation, Institute of Neuroscience and Physiology, University of Gothenburg, Goteborg, Sweden

**Keywords:** bronchopulmonary dysplasia, preterm infant, brain injury, neurological disability, neurodevelopmental impairment

## Abstract

**Objective:**

We investigated the association between bronchopulmonary dysplasia (BPD) and 3 years death or neurodevelopmental impairment (NDI) in very preterm infants without severe brain injury.

**Method:**

Our prospective cohort study recruited preterm infants who were born prior to 32 weeks of gestational age and survived in the neonatal intensive care unit until 36 weeks of corrected age. Upon reaching 3 years of age, each infant was assessed for death or NDI such as cerebral palsy, cognitive deficit, hearing loss, and blindness. Correlations between BPD and death or NDI were determined using multiple logistic regression analyses adjusted for confounding factors.

**Result:**

A total of 1,417 infants without severe brain injury who survived until 36 weeks of corrected age were initially enrolled in the study. Over the study period, 201 infants were lost to follow-up and 5 infants were excluded. Our final dataset, therefore, included 1,211 infants, of which 17 died after 36 weeks of corrected age and 1,194 were followed up to 3 years of age. Among these infants, 337 (27.8%) developed BPD. Interestingly, by 3 years of age, BPD was demonstrated to be independently associated with death or NDI, with an adjusted odds ratio of 1.935 (95% confidence interval: 1.292–2.899, *p* = 0.001), in preterm infants without severe neonatal brain injury.

**Conclusion:**

Our findings indicate that BPD is strongly associated with death or NDI in preterm infants without severe neonatal brain injury at 3 years of age. Further research is needed to understand the mechanisms linking the development of BPD with death or NDI and whether appropriate treatment of BPD may ameliorate or prevent the development of neurological complications.

## Introduction

Prematurity remains the leading cause of neonatal mortality and associated morbidities in very preterm infants (1). Although advances in neonatal medical care have significantly reduced newborn mortality rates, surviving infants still face substantial risks of neonatal morbidities and the development of long-term neurological disabilities (2, 3). While severe neonatal brain injuries, such as cystic periventricular leukomalacia (cPVL) and grade III-IV intraventricular hemorrhage (IVH), are widely recognized as contributors to adverse neurodevelopmental outcomes, they have not been observed in the majority (82.3%, 93/113) of preterm infants who later develop neurological disabilities (4). The etiology of neurodevelopmental disorders in preterm infants is, therefore, both multifactorial and poorly understood.

Preterm infants with undeveloped lungs often require supplemental oxygen therapy, which can, through hypoxia-induced oxidative stress and inflammation, contribute to the development of bronchopulmonary dysplasia (BPD) (5). Bronchopulmonary dysplasia is characterized by impaired lung function due to a reduction in alveoli and abnormal pulmonary vascular development. This can lead to short- and long-term respiratory complications that can, in turn, further exacerbate existing lung damage, resulting in recurrent wheezing episodes and even death. Among these complications, respiratory infections, particularly viral infections, are the most common cause of hospital readmission in preschool-age infants with BPD and the frequency of these infections may be attributed to the immaturity of humoral and adaptive immunity in these infants.

Interestingly, a significant increase in mortality and neurological disabilities, including retinopathy of prematurity (ROP) and abnormal brain development, among preterm infants with BPD has been demonstrated in numerous studies (6–8). A systematic review of 11 studies has reported a significant association between BPD and cerebral palsy (OR, 2.10; 95% CI: 1.57, 2.82), and this relationship is linked to the severity of BPD (9). This connection may be attributed to the brain injury resulting from recurrent episodes of hypoxemia and chronic inflammation in preterm infants affected by BPD. Furthermore, a retrospective study encompassing infants devoid of IVH and those with low-grade IVH has provided evidence that BPD might be linked to adverse neurodevelopmental outcomes, even in preterm infants without severe brain injury. Nevertheless, the precise nature of this association remains somewhat unclear due to the research limitations (10). Therefore, we, here, have conducted a prospective cohort study across two centers to further investigate the association between BPD and 3 years death or NDI in preterm infants without severe neonatal brain injury. We hypothesized that BPD could elevate the risk of adverse outcomes at 3 years in very preterm infants without severe brain injury.

## Methods

### Study design and population

This prospective cohort study recruited preterm infants with a gestational age of less than 32 weeks and who were admitted to the Third Affiliated Hospital and Children’s Hospital of Zhengzhou University between July 2015 and December 2019. BPD was defined as the continued requirement of supplemental oxygen at 36 weeks of corrected age, and BPD severity was classified in accordance with Jensen et al.’s criteria (11) by assessing the mode of respiratory support at 36 weeks of corrected age. This classification comprises Grade 1, which includes the use of nasal cannula at flow rates ≤2 L/min; Grade 2, encompassing nasal cannula at flow rates >2 L/min or non-invasive positive airway pressure; and Grade 3, involving the need for invasive mechanical ventilation. Infants who died before 36 weeks of corrected age, experienced severe brain injury (as defined below), transferred to other hospitals, had missing medical records, as well as presented with congenital cranial malformations and/or genetic or metabolic diseases, were excluded from the study. Children who developed brain injury conditions such as encephalitis, brain trauma, and epilepsy, during the follow-up period were also excluded from final analyses. This study was reviewed and approved by the Ethics Committee of the Third Affiliated Hospital of Zhengzhou University. Written informed consent was obtained from the participants’ legal guardian.

### Data collection in infants

In infants, cranial ultrasonography examinations were conducted within 3 days after birth, at 7 days, and then weekly until either death or discharge. Cerebral magnetic resonance imaging (MRI) was conducted at approximately 40 weeks of corrected age to assess the presence and severity of any potential brain injury. Severe brain injury in neonates was diagnosed by the presence of conditions such as cPVL and grade III-IV IVH, which were assessed through head ultrasound (HUS) and magnetic resonance imaging (MRI) techniques (12). The following clinical data were collected by professional neonatologists: neonatal characteristics (gestational age, birth weight, sex, small for gestational age (SGA), 5 min Apgar <4, cesarean section, twins/multiple births), maternal characteristics (pregnancy hypertension, maternal age ≥ 35 years, fetal distress, placental abruption, gestational diabetes, premature rupture of membranes, antenatal steroids), neonatal treatment and morbidities (mechanical ventilation >7 days, postnatal steroids, respiratory distress syndrome (RDS), sepsis, severe anemia, necrotizing enterocolitis (NEC), ROP, as well as the education level and monthly income of the parent(s).

Respiratory distress syndrome denoted a progressive dyspnea of preterm infants after birth (13). The diagnosis of sepsis included blood culture-confirmed sepsis and clinical sepsis. Clinical sepsis referred to the presence of severe clinical symptoms of infection requiring anti-infective therapy but with negative blood cultures (14). Severe anemia was diagnosed based on hemoglobin concentrations, respiratory status, and age (15). Retinopathy of prematurity was diagnosed according to international criteria (16). Necrotizing enterocolitis was categorized as stage II or III based on Bell’s staging criteria (17). Infants with invasive ventilator dependence beyond the first week were administered postnatal steroids, specifically, a low-dose dexamethasone regimen (18).

### Follow-up assessments and study outcomes

Regular follow-up assessments of all infants were conducted at intervals of at least 3 months following hospital discharge to monitor neurodevelopment and growth until children reached 3 years of age. Death after 36 weeks of corrected age, suffering from respiratory and neurological diseases, and readmissions due to respiratory diseases were recorded during the follow-up period. The mental development index (MDI) and motor functions at 3 years of age were evaluated according to the Bayley Scales of Infant Development II by experienced pediatric neurologists. Audiometric and visual evaluations were performed by skilled professionals at hearing screening centers and ophthalmology department. The neurologists and examiners conducting these evaluations were blinded to the treatment history of the infants.

To evaluate the association between death or NDI and the development of BPD in infants, we defined death or NDI as either death after 36 weeks of corrected age or the emergence of NDI at 3 years of age. NDI was characterized by the presence of one or more of the following conditions among the survivors: cerebral palsy (CP), MDI < 70, hearing loss, and blindness. Cerebral palsy was defined as a group of non-progressive impairments affecting the development of movement and posture, and any type and severity of CP were involved in our study (19). The MDI was based on the Bayley Scales of Infant Development II, with a score below 70 indicating severe impairment of neurodevelopment (20). Hearing impairment was defined as a partial or total loss of hearing (21). Blindness was defined as a best-corrected visual acuity worse than 20/200 (21).

### Statistical analysis

Data analysis was conducted using SPSS, version 23.0. Clinical characteristics were compared using Chi-squared tests, Fisher’s exact tests, or Kruskal-Wallis tests as appropriate. The rates of death or NDI among different groups were compared using chi-square tests or Fisher’s exact tests. Univariate analysis was conducted to understand the impact of BPD on death or NDI. A multivariable logistic regression analysis was conducted to account for the influence of potential confounding factors on death or NDI. Subgroup analysis was performed using the Mantel–Haenszel test. A value of *p* < 0.05 indicated statistical significance.

## Results

### Population characteristics

**Figure 1:**
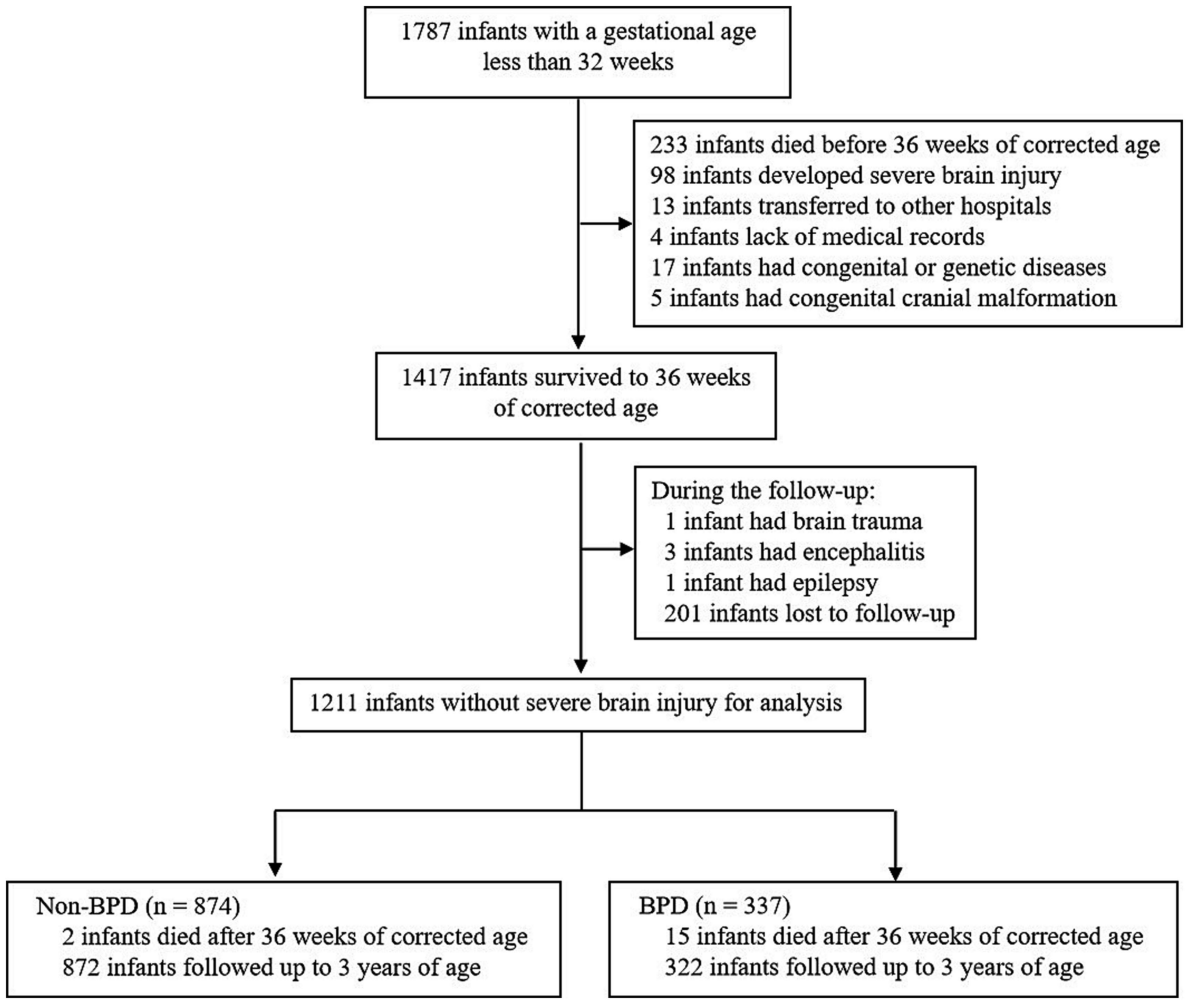
Study schematic. The flow chart showing the number of infants who were screened for analysis and followed up to 3 years of age.

A total of 1,787 infants with a gestational age of less than 32 weeks were initially enrolled in this study. Among them, 1,417 infants survived until 36 weeks of corrected age without developing exclusionary criteria such as the development of severe brain injury (Figure 1). During the follow-up period, 201 infants were lost to follow-up at 3 years of age and 5 infants developed exclusionary criteria (1 with brain trauma, 3 with encephalitis, and 1 with epilepsy). Thus, a total of 1,211 infants were included in the final analyses, of which 17 died after 36 weeks of corrected age and 1,194 were followed up to 3 years of age (Figure 1). Out of the 1,211 infants included in the study, 337 (27.8%) of them developed BPD. Within this group of 337 infants with BPD, 75 (22.3%) were categorized as having Grade 2 & 3 BPD. All comprehensive neonatal characteristics, maternal characteristics, and neonatal morbidities are summarized in Table 1.

**Table 1:** Clinical characteristics between BPD and non-BPD preterm infants without severe brain injury.

	Non-BPD (*n* = 874)	BPD (*n* = 337)	*p*-value
Neonatal characteristics			
Gestational age, (weeks, median)	30.0 (1.9)	29.1 (1.6)	0.000
Birth weight, (g, median)	1,315 (336)	1,150 (300)	0.000
Male, *n* (%)	477 (54.6)	212 (62.9)	0.009
SGA, *n* (%)	27 (3.1)	10 (3.0)	0.912
5 min Apgar <4, *n* (%)	23 (2.6)	22 (6.5)	0.001
Cesarean section births, *n* (%)	563 (64.4)	212 (62.9)	0.624
Two/Multiple births, *n* (%)	247 (28.3)	92 (27.3)	0.738
Maternal characteristics			
Pregnancy hypertension, *n* (%)	131 (15.0)	63 (18.7)	0.115
Maternal age ≥ 35 years, *n* (%)	184 (21.1)	66 (19.6)	0.572
Fetal distress, *n* (%)	140 (16.0)	54 (16.0)	0.998
Placental abruption, *n* (%)	57 (6.5)	23 (6.8)	0.849
Gestational diabetes, *n* (%)	75 (8.6)	20 (5.9)	0.125
PROM, *n* (%)	180 (20.6)	56 (16.6)	0.117
Antenatal steroids	708 (82.8)	265 (78.9)	0.114
Neonatal treatment and morbidities			
MV > 7 days, *n* (%)	62 (7.1)	94 (27.9)	0.000
Postnatal steroids	12 (1.4)	24 (7.1)	0.000
RDS, *n* (%)	778 (89.0)	314 (93.2)	0.029
Sepsis, *n* (%)	140 (16.0)	130 (38.6)	0.000
Severe anemia, n (%)	386 (44.2)	211 (62.6)	0.000
NEC, *n* (%)	30 (3.4)	16 (4.7)	0.283
ROP, *n* (%)	36 (4.1)	26 (7.7)	0.011
Parent’s education level			0.087
Middle school, *n* (%)	241 (27.6)	75 (22.3)	
High school, *n* (%)	324 (37.1)	145 (43)	
University, *n* (%)	309(35.4)	117 (34.7)	
Parent’s monthly income			0.148
<5,000 yuan, *n* (%)	166 (19)	56 (16.6)	
5,000–10,000 yuan, *n* (%)	459 (52.5)	198 (58.8)	
>10,000 yuan, *n* (%)	249 (28.5)	83 (24.6)	

BPD, bronchopulmonary dysplasia; SGA, small for gestational age; PROM, premature rupture of membranes; MV, mechanical ventilation; RDS, respiratory distress syndrome; NEC, necrotizing enterocolitis; ROP, retinopathy of prematurity.

Infants with BPD and those without BPD were observed to share similar maternal and neonatal characteristics such as small for gestational age (SGA), cesarean section births, twin/multiple births, pregnancy hypertension, maternal age ≥ 35 years, fetal distress, placental abruption, gestational diabetes, premature rupture of membranes, antenatal steroids, NEC, as well as the education level and monthly income of the parent(s). However, when compared to infants without BPD, infants with BPD exhibited significantly lower gestational age (*p* = 0.000), birth weight (*p* = 0.000), as well as higher rates of being male (*p* = 0.009), 5-min Apgar score < 4 (*p* = 0.001), needing mechanical ventilation exceeding 7 days (*p* = 0.000), postnatal steroids treatment (*p* = 0.000), and developing RDS (*p* = 0.029), sepsis (*p* = 0.000), severe anemia (*p* = 0.000), and ROP (*p* = 0.011) (Table 1).

During the follow-up period until 3 years of age, infants with BPD exhibited a significantly higher rate of recurrent hospitalization due to respiratory infections compared to infants without BPD (82 out of 322, 25.5% vs. 95 out of 872, 10.9%, *p* = 0.000). Further, 6 infants (1.9%) with BPD and 3 (0.9%) without BPD developed asthma, and 36 infants (11.2%) with BPD and 43 (4.9%) without BPD received neurodevelopmental rehabilitation training.

### The impact of BPD on death or NDI of preterm infants at 3 years of age

Univariate analysis revealed that, by 3 years of age, infants with BPD exhibited significantly higher rates of CP (4.3% vs. 0.7%, *p* = 0.000), MDI < 70 (10.2% vs. 6.4%, *p* = 0.025), NDI (15.5% vs. 8.3%, *p* = 0.000), death (4.5% vs. 0.2%, *p* = 0.000), and death or NDI (19.3% vs. 8.5%, *p* = 0.000) compared to infants without BPD. However, observations of deafness (1.2% vs. 1.1%, *p* = 1.000) and blindness (1.9% vs. 0.5%, *p* = 0.045) were similar between the two infant groups (Table 2).

**Table 2:** Unadjusted death or NDI at 3 years of age between BPD and non-BPD preterm infants without severe brain injury.

	Non-BPD, *n*/Total (%)	BPD, *n*/Total (%)	*p*-value
CP	6/872 (0.7)	14/322 (4.3)	0.000
MDI <70	56/872 (6.4)	33/322 (10.2)	0.025
Deafness	10/872 (1.1)	4/322 (1.2)	1.000
Blindness	4/872 (0.5)	6/322 (1.9)	0.045
NDI	72/872 (8.3)	50/322 (15.5)	0.000
Death	2/874 (0.2)	15/337 (4.5)	0.000
Death or NDI	74/874 (8.5)	65/337 (19.3)	0.000

BPD, bronchopulmonary dysplasia; CP, cerebral palsy; MDI, mental development index; NDI, neurodevelopmental impairment.

Subsequently, we performed a multivariable logistic regression analysis to account for potential confounding variables, including gestational age, birth weight, sex, duration of mechanical ventilation exceeding 7 days, sepsis, severe anemia, and postnatal steroids, in order to assess their impact on death or NDI. Notably, even after adjusting for these confounding factors, the results demonstrated that BPD maintained a significant association with an elevated risk of adverse outcomes in preterm infants who did not exhibit severe brain injury and reached the age of 3 [adjusted odds ratio (aOR) = 1.935, 95% confidence interval (CI): 1.292–2.899, *p* = 0.001]. Comprehensive details of the results from both univariate and multivariable logistic regression analyses are presented in Tables 2, 3, respectively.

**Table 3:** Multivariable logistic regression analysis on the impact of BPD on death or NDI in preterm infants without severe brain injury at 3 years of age.

	*B*	SE	Wald	df	aOR (95% CI)	*p*-value
Gestational age	−0.168	0.083	4.152	1	0.845 (0.719–0.994)	0.042
Birth weight	0.000	0.000	0.405	1	1.000 (0.999–1.001)	0.524
Male	0.304	0.195	2.422	1	1.355 (0.924–1.986)	0.120
MV > 7 days	0.205	0.268	0.588	1	1.228 (0.726–2.075)	0.443
Sepsis	0.367	0.208	3.091	1	1.443 (0.959–2.171)	0.079
Severe anemia	0.358	0.196	3.328	1	1.430 (0.974–2.101)	0.068
Postnatal steroids	−0.057	0.479	0.014	1	0.945 (0.370–2.415)	0.906
BPD	0.660	0.206	10.241	1	1.935 (1.292–2.899)	0.001
Constant	1.832	2.263	0.655	1	6.245	0.418

CI, confidence interval; SE, standard error; aOR, adjusted odds radio; MV, mechanical ventilation; BPD, bronchopulmonary dysplasia; NDI, neurodevelopmental impairment.

To further elucidate the effect of BPD severity on death or NDI, we conducted a multivariable logistic regression analysis based on BPD severity. This analysis indicated that Grade 2 & 3 BPD was significantly associated with adverse outcomes (aOR = 3.407, 95% CI: 1.923–6.037, *p* = 0.000), whereas Grade 1 BPD exhibited a less pronounced association (aOR = 1.549, 95% CI: 0.988–2.429, *p* = 0.057) (Supplementary Table 1).

### Effect of gestational age, birth weight, and sex on the association of BPD with death or NDI

Subgroup analyses were conducted based on three strata of gestational age (<28 weeks, 28–29/6 weeks, and 30–32 weeks), three strata of birth weight (<1,000 g and 1,000–1,499 g, and ≥ 1,500 g), and two strata of sex (female and male). The results showed that BPD significantly increased the incidence of death or NDI in preterm infants across two gestational age strata (<28 weeks and 28–29/6 weeks), two birth weight strata (<1,000 g and 1,000–1,499 g), and in both sex strata (male and female). However, all the interactions were not statistically significant (*p* > 0.05) (Table 4), thus suggesting that gestational age, birth weight, and sex do not have a significant interactive effect on the impact of BPD on death or NDI in preterm infants without severe brain injury. The results from subgroup analyses are presented in Table 4.

**Table 4:** Subgroup interaction analysis on the impact of BPD on death or NDI in preterm infants without severe brain injury at 3 years of age.

Subgroups	Non-BPD	BPD	OR(95% CI)	Interaction *p-*value
Gestational age				
< 28 weeks	5/51 (9.8)*	15/56 (26.8)	3.366 (1.125–10.073)	
28–29^6/7^ weeks	37/367 (10.1)***	40/192 (20.8)	2.347 (1.443–3.818)	
30–32 weeks	32/456 (7.0)	10/89 (11.2)	1.677 (0.793–3.549)	
Total			2.583 (1.802–3.705)	0.563
Birth weight				
< 1,000 g	6/67 (9.0)*	14/62 (22.6)	2.965 (1.060–8.293)	
1,000–1,499 g	51/549 (9.3)***	48/257 (18.7)	2.243 (1.465–3.434)	
≥ 1,500 g	17/258 (6.6)	3/18 (16.7)	2.835 (0.747–10.759)	
Total			2.583 (1.802–3.705)	0.853
Sex				
Male	47/477 (9.9)***	44/212 (20.8)	2.396 (1.531–3.751)	
Female	27/397 (6.8)**	21/125 (16.8)	2.767 (1.503–5.094)	
Total			2.583 (1.802–3.705)	0.709

BPD, bronchopulmonary dysplasia; OR, odds radio; CI, confidence interval. NDI, neurodevelopmental impairment. Mantel–Haenszel test was used for interaction analysis. *p < 0.05, **p < 0.01, ***p < 0.001.

## Discussion

A comprehensive understanding of the etiology of neurodevelopmental disorders in preterm infants is necessary to improve the survival rates and long-term quality of life of these infants. Here, using a large prospective cohort, we demonstrate that BPD in very preterm infants without severe neonatal brain injury is independently associated with an increased risk of death or NDI by 3 years of age.

BPD, as a chronic pulmonary disease, is associated with several neonatal morbidities, such as ROP and abnormal brain development, in infants (22, 23), but the mechanisms underlying this association are unclear (24). Several studies have suggested that lung-brain injury interactions may involve complex processes such as inflammation, oxidative stress, neurodegeneration, vascular formation, and structural remodeling (25–27). Indeed, a shared pathway between lung and brain injuries has been implicated by the observation that hyperoxia-induced injuries elicit similar oxidative stress responses in the immature lung and brain of infants (5). Further, prolonged exposure to oxygen can trigger lung inflammation and disrupt lung development, as well as contribute to abnormal white matter development in infants with immature brains due to the inflammatory activation of oligodendrocytes (25, 28). Exposure to hyperoxic conditions may also increase the production of reactive oxygen species, resulting in damage to immature organs (29). Importantly, hyperoxia-induced oxidative stress and inflammation in the lungs of preterm infants can lead to the development of BPD (30). Thus, infants with BPD experience both impaired lung growth and an increased risk of neurodevelopmental abnormalities (31, 32). Conversely, it is possible that abnormal brain development occurs as a secondary injury process resulting from abnormal lung development via the lung-brain axis. Children with BPD exhibit persistent structural abnormalities in their lungs (e.g., abnormal vessel distribution, reduced vessel densities, and alveolar simplification) and respiratory dysfunction (33), which are associated with altered white matter development and may exert long-term negative effects on the neurodevelopment of infants with immature brains (34). Overall, our findings show that BPD is a predictive factor for death or NDI in preterm infants, but additional studies are needed to discern the exact mechanisms and pathways mediating the interactions between lung and brain abnormalities/injuries in this vulnerable population.

Retinopathy of prematurity is a common occurrence in extremely preterm infants with BPD and has been linked to neurological disability (35, 36). Accordingly, our findings showed a higher incidence of severe ROP in infants with BPD compared to those without BPD. The development of ROP involves several complex factors that include relative hyperoxia, which damages the immature retina in infants with BPD (37). Further, concurrent inflammation in the lungs and eyes of infants receiving oxygen support may potentially contribute to the development of both BPD and ROP (38). To reduce the occurrence of ROP and improve neurological outcomes, it is crucial to monitor oxygen levels in infants with BPD using innovative technologies and methods.

Several factors may contribute to the association between BPD in preterm infants and the development of death or NDI later in life. For instance, following hospital discharge, infants with BPD face a heightened risk of rehospitalization due to factors such as compromised nutrition, growth and developmental abnormalities, weakened immune system, and heightened susceptibility to respiratory viral infections (39, 40), the latter of which was observed in our study population. These risks may contribute to worsened neurological outcomes as well as increased mortality and healthcare costs. Interestingly, our findings revealed that, while the probability of death or NDI was significantly higher in male infants as well as those with lower gestational age and birth weight, the impact of BPD on death or NDI was consistent across different gestational ages, birth weights, and sex. This indicates that the association between BPD and death or NDI is not influenced by gestational age, birth weight, and sex. Together, these findings suggest that prioritizing both home care, including nutritional support, and hospital-based interventions for infants diagnosed with BPD are essential to mitigate the risk of developing death or NDI later in life.

It is important to note the limitations of our study. First, the follow-up period only extended until the age of 3, which prevented the identification of poor neurological outcomes that may emerge later in life (41). Consequently, future efforts should focus on extending the follow-up period, potentially up to school age or beyond, to better understand the impact of BPD on the long-term neurological development of preterm infants. Second, we did not collect information regarding the initiation and duration of home oxygen therapy, which is a common at-home treatment for promoting the development and growth of preterm infants with BPD (42). Therefore, our data do not account for any differences in home treatment.

## Conclusion

Our findings demonstrate that BPD in very preterm infants without severe brain injury is strongly associated with increased risk of mortality and neurological disabilities at 3 years of age. This identifies an urgent need to monitor the neurological development of preterm infants with BPD as well as develop treatment strategies that can help prevent or mitigate death or NDI in preterm infants with BPD. To address these challenges, further research is warranted to investigate the underlying pathophysiological mechanisms that connect lung injury and neurological disability in very preterm infants.

## Data availability statement

The raw data supporting the conclusions of this article will be made available by the authors, without undue reservation.

## Ethics statement

The studies involving humans were approved by the Ethics Committee of the Third Affiliated Hospital of Zhengzhou University. The studies were conducted in accordance with the local legislation and institutional requirements. Written informed consent for participation in this study was provided by the participants' legal guardians/next of kin.

## Author contributions

WL: Writing – original draft, Writing – review & editing, Conceptualization, Data curation, Formal analysis, Investigation, Methodology, Visualization. YW: Conceptualization, Data curation, Formal analysis, Investigation, Methodology, Visualization, Writing – original draft, Writing – review & editing. JS: Conceptualization, Data curation, Formal analysis, Investigation, Methodology, Validation, Writing – original draft, Writing – review & editing. CZha: Conceptualization, Data curation, Investigation, Methodology, Writing – review & editing. YX: Conceptualization, Formal analysis, Methodology, Validati on, Visualization, Writing – review & editing. FX: Conceptualization, Formal analysis, Investigation, Methodology, Supervision, Validation, Writing – review & editing. XW: Conceptualization, Formal analysis, Methodology, Supervision, Visualization, Writing – review & editing. CZhu: Conceptualization, Data curation, Formal analysis, Funding acquisition, Investigation, Methodology, Project administration, Supervision, Validation, Writing – review & editing.
