# Supplementary material for: Association between bronchopulmonary dysplasia and death or neurodevelopmental impairment at 3 years in preterm infants without severe brain injury
Source: Front Neurol. 2023 Nov 15;14:1292372. doi: 10.3389/fneur.2023.1292372 (PMC10684711; doi:10.3389/fneur.2023.1292372)
Supplement: Supplementary file 1 [file Table_1.docx]

**Supplementary Table 1. Multivariable logistic regression analysis the influence of BPD severity on adverse outcomes in preterm infants without severe brain injury**

|  | **Grade 1 BPD (n = 262)** | | **Grade 2 & 3 BPD (n = 75)** | |
| --- | --- | --- | --- | --- |
|  | **aOR (95% CI)** | ***P*-value** | **aOR (95% CI)** | ***P*-value** |
| CP | 4.101 (1.335-12.596) | 0.014 | 8.234 (2.334-29.053) | 0.001 |
| MDI <70 | 1.450 (0.873-2.408) | 0.151 | 1.921 (0.907-4.072) | 0.088 |
| Deafness | 0.701 (0.153-3.220) | 0.648 | 2.394 (0.515-11.137) | 0.266 |
| Blindness | 0.372 (0.037-3.756) | 0.402 | 8.587 (2.106-35.002) | 0.003 |
| NDI | 1.187 (0.732-1.923) | 0.487 | 3.059 (1.680-5.567) | 0.000 |
| Death | 10.019 (2.071-48.479) | 0.004 | 12.162 (2.048-72.232) | 0.006 |
| Death or NDI | 1.549 (0.988-2.429) | 0.057 | 3.407 (1.923-6.037) | 0.000 |

Non-BPD group was set as the reference group. BPD, bronchopulmonary dysplasia; CP, cerebral palsy; MDI, mental development index; aOR, adjusted odds radio; CI, confidence interval; NDI: neurodevelopmental outcome.
